# The mediating role of body mass index in the association between unprocessed or minimally processed foods and gallstones

**DOI:** 10.3389/fnut.2025.1589805

**Published:** 2025-06-23

**Authors:** Chenyu Jiang, Luqi Zhu, Xiaosheng Teng, Hongxun Wen, Zhenjun Yu, Weiwei Yang, Yaojian Shao

**Affiliations:** ^1^Department of Geriatric, Taizhou Central Hospital (Taizhou University Hospital), Taizhou, Zhejiang, China; ^2^Department of Critical Care Medicine, Taizhou Hospital of Zhejiang Province Affiliated to Wenzhou Medical University, Taizhou, Zhejiang, China; ^3^Department of Gastroenterology, Taizhou Central Hospital (Taizhou University Hospital), Taizhou, Zhejiang, China

**Keywords:** unprocessed or minimally processed foods, ultra-processed foods, NOVA, gallstones, body mass index, mediation, NHANES

## Abstract

**Background:**

The extent of food processing significantly impacts human health, with ultra-processed foods (UPFs) linked to numerous adverse health outcomes. In contrast, research on unprocessed or minimally processed foods (MPFs) and their association with gallstones remains scarce. This study aimed to investigate the relationship between MPF intake and gallstones in U.S. adults.

**Methods:**

We conducted a cross-sectional analysis using data from the National Health and Nutrition Examination Survey (NHANES, 2017–2023). MPF intake was assessed according to the NOVA classification system. Survey-weighted logistic regression, restricted cubic spline models, and mediation analyses were employed to evaluate the association between MPF consumption and gallstones disease.

**Results:**

Among 11,779 U.S. adults, 1,303 cases of gallstones disease were identified (weighted prevalence: 9.8%). Elevated percentage contribution of MPF was significantly associated with a reduced likelihood of gallstones [model 1, odds ratio (OR): 0.40, 95% confidence interval (CI): 0.21–0.78], and this inverse relationship persisted after full adjustment (model 3; OR: 0.28, 95% CI: 0.09–0.84). Compared to the lowest quartile (Q1), the highest quartile (Q4) of MPF consumption showed significantly lower odds of gallstones (OR: 0.72, 95% CI: 0.53–0.98). A non-linear, inverted U-shaped relationship was observed between MPF intake and gallstones (overall *p* < 0.001; non-linear *p* = 0.031). Mediation analysis indicated that the body mass index (BMI) partially mediated this association. No significant associations were found between other NOVA food groups, including UPF, and gallstones disease.

**Conclusion:**

Higher MPF consumption is associated with a lower risk of gallstones disease, with BMI partially mediating this relationship.

## 1 Introduction

Gallstones disease, a prevalent hepatobiliary disorder, represents a significant global health burden with substantial economic implications. The prevalence of gallstones varies across region, with estimates ranging from 10% to 20% in America, Europe, and other developed countries, and 5% reported in certain parts of Asia (1, 2). While most adult patients are asymptomatic, about 25% experience symptoms and complications, including cholecystitis, pancreatitis, and cholangitis, which often necessitate surgical treatment for effective management (3–6). The high prevalence of gallstones disease and its associated complications, along with the necessary treatments and surgical interventions, impose a substantial medical burden on patients and significantly elevate healthcare costs at the societal level (7). This trend is anticipated to intensify, as the prevalence of gallstones disease has risen significantly and has doubled over the past few decades (8). Therefore, investigating primary prevention strategies for gallstones disease, such as dietary modifications and lifestyle changes, may offer significant benefits in reducing its prevalence and alleviating associated healthcare costs.

Gallstones formation is a multifactorial process influenced by various factors, including age, sex, obesity, sedentary lifestyle, dietary factors, and inflammatory response (9). Diet represents a practical and accessible approach to disease prevention (10) and has been demonstrated to modulate the progression of gallstones disease (11, 12). The high consumption of carbohydrates, caloric diet, and glycemic load were associated with higher risk, while high levels of fiber, vegetable, and fruit consumption were protective factors (13–15). Over the past two decades, there has been a global shift toward increased consumption of ultra-processed foods (UPF) and a corresponding decline in the intake of unprocessed or minimally processed foods (MPFs) (16). These foods are classified under the NOVA system, which categorizes food products based on their level of industrial processing (17). Excessive consumption of UPF has been shown to be associated with an increased risk of gallstones disease (18). MPF, which occupy the opposite end of the food processing spectrum, defined as foods are consumed in their natural state or altered by methods designed to preserve their nutritional content. The high dietary fiber composition of MPFs may reduce gallstone formation propensity by promoting bile acid enterohepatic circulation to mitigate biliary cholesterol supersaturation (19). While numerous studies have focused on the association between UPF and chronic diseases, research exploring the relationship between MPF and disease remains scarce in comparison. In particular, the association between MPF and gallstones disease is still unclear.

In this study, we performed a cross-sectional analysis utilizing data from the National Health and Nutrition Examination Survey (NHANES) to investigate the association between MPF consumption and gallstones disease among US adults. Additionally, we explored the potential mediating effects of body mass index (BMI) on this association.

## 2 Materials and methods

### 2.1 Study population

**FIGURE 1:**
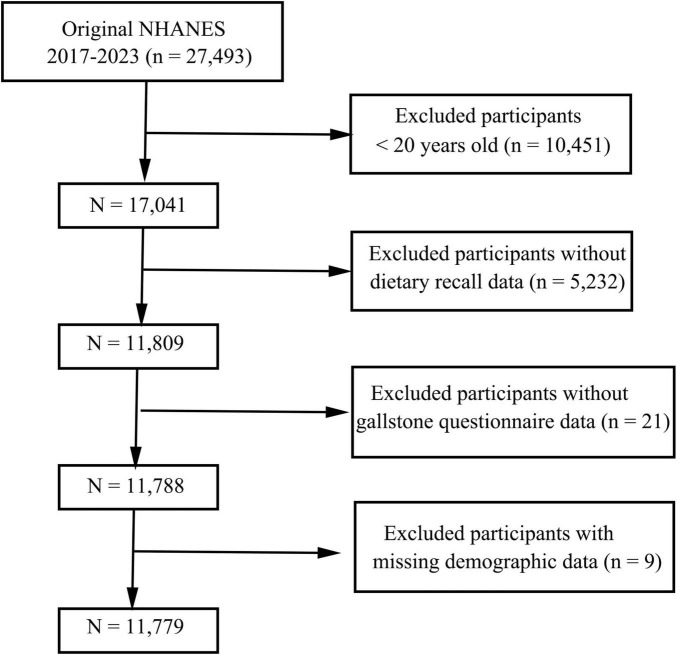
Flowchart of participant selection.

This cross-sectional study utilized data from the NHANES, a nationally representative survey employing a complex, stratified, multistage probability sampling design. Written informed consent was obtained from all participants or their guardians prior to data collection. Data from three consecutive NHANES cycles (2017–2023) were analyzed. The initial dataset included 27,493 participants. Exclusion criteria were as follows: individuals aged < 20 years (*n* = 10,451), those with incomplete dietary recall or gallstones questionnaire data (*n* = 5,253), and participants lacking demographic information (*n* = 9). After exclusions, the final analytical sample consisted of 11,779 participants, as illustrated in Figure 1.

### 2.2 Assessment of food consumption

Dietary intake data were collected through 24-h dietary recalls conducted during the NHANES cycles (2017–2023). Based on the NOVA classification system, food items were categorized into four mutually exclusive groups according to the extent and purpose of food processing: UPFs, processed foods (PF), processed culinary ingredients (PCI), and unprocessed or MPF. The primary exposure variables in this study were the percentage contribution of MPF to total daily energy intake and the quartiles of MPF consumption (kcal/d).

### 2.3 Definition of gallstones

Gallstones presence was identified through participants’ responses to the question, “Has a doctor ever told you that you have gallstones?” Participants who answered “Yes” were categorized as having gallstones, while those who answered “No” were categorized as not having gallstones.

### 2.4 Study covariates

Potential confounding factors influencing gallstones were carefully considered based on the reference. Additional participant data extracted from the NHANES database for this study included ethnicity (Mexican American, non-Hispanic White, other Hispanic, non-Hispanic Black, and other), age (years), sex (male or female), family poverty-to-income ratio (PIR), education (high school, and below and above high school), intake of energy (kcal), BMI (kg/m^2^), smoker/non-smoker [non-smoker (<100 lifetime cigarettes or more than this threshold but not a current smoker), smoker (>100 lifetime cigarettes and current smoker)], drinker/non-drinker [non-drinker (<12 drinks over lifetime or 12+ per year but none in the past year), drinker (within previous 12 months)], as well as presence of hypertension, diabetes mellitus (DM), and hyperlipidemia. DM was defined based on self-reported diagnosis, use of insulin or antidiabetic medication, FBG ≥ 126 mg/dl, HbA1c ≥ 6.5%, or serum glucose ≥ 200 mg/dl 2 h after loading with 75 g oral glucose. The determination of hypertensive status was based on a systolic blood pressure level of 140 mmHg or higher and/or a diastolic blood pressure level of 90 mmHg or higher, a history of antihypertensive treatment, or a diagnosis of hypertension that was self-reported. Hyperlipidemia was defined as triglyceride (TG) levels ≥ 150 mg/dl (1.7 mmol/L), total cholesterol (TC) ≥ 200 mg/dl (5.18 mmol/L), low-density lipoprotein (LDL) ≥ 130 mg/dl (3.37 mmol/L), or high-density lipoprotein (HDL) < 40 mg/dl (1.04 mmol/L) in men and < 50 mg/dl (1.30 mmol/L) in women. Individuals using cholesterol-lowering medications were also classified as hyperlipidemic (20).

### 2.5 Statistical analyses

To account for the multistage sampling design of NHANES, sample weighting was leveraged for all analyses. Categorical variables are expressed as counts (weighted percentages) and assessed using Chi-square tests, while continuous variables are shown as means ± SE and analyzed using Student’s *t* tests. Weighted univariate and multivariate logistic regression were utilized to assess links between MPF intake (kcal/d) and gallstones, generating odds ratios (ORs) with 95% confidence intervals (CIs). Model 1 received no adjustment, model 2 had adjustments for age, sex, education, ethnicity, and PIR, and model 3 included further adjustments for BMI, smoking status, alcohol consumption, DM, hypertension, and hyperlipidemia. Restricted cubic spline (RCS) models were utilized for assessing non-linear associations. Subgroup analyses were conducted in terms of age (<60, ≥60), sex, ethnicity, education, BMI (<25, 25–30, and >30 kg/m^2^), DM, hypertension, hyperlipidemia, smoking, and drinking. Mediation analysis was performed using ‘mediation” in R with 1,000 bootstrap iterations to examine how BMI mediates the relationship between the daily percentage intakes of MPF and gallstone disease. Analyses were conducted in R (v 4.2.2), with *p* < 0.05 indicating significance.

## 3 Results

### 3.1 Participants

Overall, 11,779 participants were enrolled in this study and categorized into quartiles based on MPF intake (kcal/d). The weighted prevalence of gallstones disease in this population was approximately 9.8%. In comparison with those in the lowest quartile (Q1), fewer participants in the highest quartile (Q4) were identified as gallstones. Additionally, higher MPF intake were related to greater proportions of males, individuals of Mexican American, other Hispanic, and other race, higher education status, and greater PIR. Participants in Q4 also consumed more energy, had lower BMI, a lower prevalence of DM and hypertension, fewer smokers and alcohol consumers compared to those in Q1. A summary of baseline characteristics is provided in Table 1.

**TABLE 1:** Basic participant characteristics classified according to quartiles of MPF consumption (kcal/d).

		MPF				
Variable	Total	Q1 (0–8 kcal)	Q2 (9–122 kcal)	Q3 (123–274 kcal)	Q4 (275–4,037 kcal)	*p-*Value
Age (years)	48.84 (0.41)	45.63 (0.69)	50.05 (0.60)	49.98 (0.54)	49.80 (0.64)	<0.0001
Sex (%)						<0.0001
Female	6,316 (52.07)	1,542 (48.37)	1,837 (57.88)	1,559 (53.89)	1,378 (47.33)	
Male	5,463 (47.93)	1,415 (51.63)	1,252 (42.12)	1,245 (46.11)	1,551 (52.67)	
Ethnicity (%)						<0.0001
Mexican American	1,146 (7.80)	169 (5.13)	245 (5.34)	312 (10.63)	420 (10.90)	
Non-Hispanic Black	2,425 (11.27)	840 (14.90)	600 (9.69)	526 (11.29)	459 (9.13)	
Non-Hispanic White	5,361 (61.66)	1,373 (63.50)	1,577 (67.70)	1,258 (58.39)	1,153 (55.59)	
Other Hispanic	1,211 (8.87)	237 (7.36)	303 (9.01)	298 (8.59)	373 (10.65)	
Other race	1,636 (10.40)	338 (9.11)	364 (8.26)	410 (11.10)	524 (13.72)	
Education (%)						<0.0001
Less than high school	673 (3.09)	99 (1.83)	135 (2.23)	197 (3.99)	242 (4.61)	
High school	3,651 (31.31)	1,134 (37.18)	901 (30.21)	792 (28.93)	824 (28.53)	
More than high school	7,455 (65.60)	1,724 (60.99)	2,053 (67.55)	1,815 (67.08)	1,863 (66.86)	
PIR	3.15 (0.06)	2.97 (0.07)	3.24 (0.08)	3.17 (0.08)	3.22 (0.08)	<0.001
Energy intake (kcal)	2,090.97 (12.58)	1,987.36 (24.34)	1,991.01 (28.15)	2,031.93 (17.19)	2,385.64 (35.96)	<0.0001
Body mass index (BMI, kg/m^2^)	29.78 (0.17)	31.05 (0.17)	29.85 (0.21)	29.61 (0.25)	28.46 (0.30)	<0.0001
<25	2,921 (25.83)	620 (20.80)	735 (24.56)	707 (27.40)	859 (32.17)	
25–30	3,820 (33.07)	837 (31.09)	971 (34.25)	943 (32.58)	1,069 (35.37)	
>30	4,906 (40.36)	1,463 (48.11)	1,343 (41.19)	1,121 (40.02)	979 (32.45)	
DM (%)						0.14
No	9,378 (84.13)	2,360 (84.61)	2,449 (84.85)	2,195 (83.74)	2,374 (87.01)	
Yes	2,291 (14.81)	575 (15.39)	615 (15.15)	578 (16.26)	523 (12.99)	
Hypertension (%)						0.05
No	6,402 (61.86)	1,590 (59.98)	1,642 (61.50)	1,491 (61.35)	1,679 (64.92)	
Yes	5,376 (38.13)	1,367 (40.02)	1,447 (38.50)	1,312 (38.65)	1,250 (35.08)	
Hyperlipidemia (%)						<0.001
No	4,317 (38.44)	1,121 (40.09)	1,077 (35.88)	1,027 (41.80)	1,092 (43.32)	
Yes	7,084 (57.55)	1,722 (59.91)	1,923 (64.12)	1,670 (58.20)	1,769 (56.68)	
Smoke (%)						<0.0001
No	9,969 (85.55)	2,313 (80.11)	2,647 (87.37)	2,451 (87.37)	2,558 (87.94)	
Yes	1,799 (14.34)	640 (19.89)	441 (12.63)	350 (12.63)	368 (12.06)	
Alcohol user (%)						0.12
No	995 (7.23)	186 (7.22)	233 (8.80)	272 (9.87)	304 (10.14)	
Yes	8,120 (73.61)	2,137 (92.78)	2,138 (91.20)	1,863 (90.13)	1,982 (89.86)	
Gallstone (%)						0.004
No	10,476 (90.20)	2,609 (89.41)	2,711 (88.82)	2,477 (90.19)	2,679 (92.75)	
Yes	1,303 (9.80)	348 (10.59)	378 (11.18)	327 (9.81)	250 (7.25)	

MPF, unprocessed or minimally processed foods; BMI, body mass index; PIR, poverty income ratio; DM, diabetes mellitus.

### 3.2 Association of the MPF intake and gallstones

**FIGURE 2:**
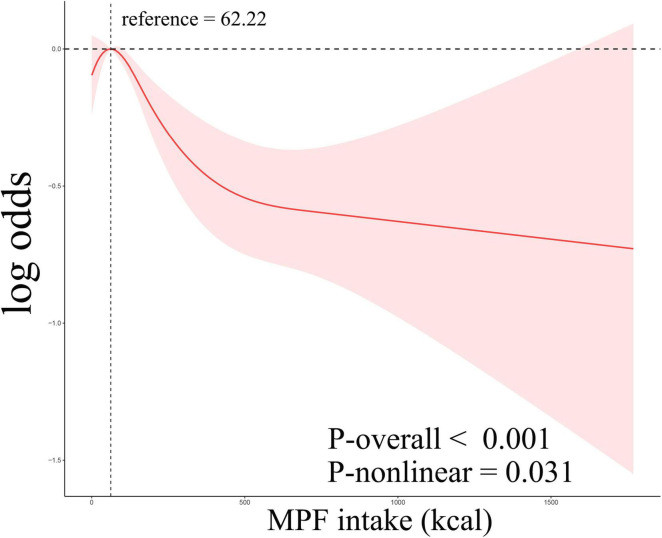
Restricted cubic spline curves corresponding to the association between MPF consumption and gallstones.

Weighted logistic regression analyses were used when assessing relationships between MPF intake and gallstones (Table 2). In the unadjusted model 1, higher percentage contribution of MPF were linked with reduced likelihood of gallstones (OR: 0.40, 95% CI: 0.21–0.78). Compared to Q1, participants in Q4 (OR: 0.66, 95% CI: 0.52–0.84) exhibited markedly lower odds of gallstones. After adjustment for age, sex, education, ethnicity, and PIR in model 2, the inverse relationship between percentage contribution of MPF and gallstones was still significant (OR: 0.21, 95% CI: 0.10–0.46). Compared with Q1, the odds of gallstones in Q3 (OR: 0.76, 95% CI: 0.60–0.95), Q4 (OR: 0.60, 95% CI: 0.47–0.78) was significantly reduced by 24%, and 40%, respectively. We further adjusted for BMI, smoking status, alcohol consumption, DM, hypertension, and hyperlipidemia in model 3 and found that the negative association was still observed (OR: 0.28, 95% CI: 0.09–0.84). Compared to Q1, significantly reduced odds of gallstones were observed only in Q4 (OR: 0.72, 95% CI: 0.53–0.98). The overall trend analysis yielded a *p*-value of 0.01. Higher percentage contribution of PF was associated with increased likelihood of gallstones in model 1 (OR: 1.76, 95% CI: 1.18–2.64; Supplementary Table 1). However, no significant association was observed between the intake of other food components, including UPF, PF, and PCI, and gallstones in model 2 and model 3 (Supplementary Table 1). To investigate potential non-linearity, RCS models were employed. As illustrated in Figure 2, MPF intake showed a statistically significant non-linear association with gallstones (overall *p*-value < 0.001, non-line *p*-value=̃ 0.031), displaying an inverted U-shaped pattern. At MPF intake exceeded 62.22 kcal/d, the rate of gallstones trended downward with increases in MPF intake. When these levels below 62.22 kcal/d, the rate of gallstones gradually trended upward.

**TABLE 2:** Association of the MPF consumption and gallstones by logistic regression.

	Model 1		Model 2		Model 3	
Variable	OR (95% CI)	*p*-Value	OR (95% CI)	*p*-Value	OR (95% CI)	*p*-Value
MPF (% of total energy intake)	0.40 (0.21–0.78)	0.01	0.21 (0.10–0.46)	<0.001	0.28 (0.09–0.84)	0.02
Quartile of MPF intake (kcal)						
Q1	Ref.		Ref.		Ref.	
Q2	1.06 (0.84–1.35)	0.61	0.87 (0.68–1.12)	0.26	0.99 (0.72–1.35)	0.95
Q3	0.92 (0.72–1.17)	0.47	0.76 (0.60–0.95)	0.02	0.84 (0.63–1.13)	0.25
Q4	0.66 (0.52–0.84)	0.001	0.60 (0.47–0.78)	<0.001	0.72 (0.53–0.98)	0.04
*p* for trend		<0.001		<0.001		0.02

MPF, unprocessed or minimally processed foods; OR, odds ratio; CI, confidence interval. Model 1 did not adjust for covariates. Model 2 adjusted for age, sex, education, ethnicity, and PIR. Model 3 adjusted for age, sex, education, ethnicity, PIR, BMI, smoking status, alcohol consumption, DM, hypertension, and hyperlipidemia.

### 3.3 Subgroup analysis

Stratified analyses were performed across various subgroups. Table 3 illustrates that the inverse relationship between MPF intake and gallstones was apparent in the age (<60), sex (female), educational level (<high school and >high school) subgroups. This relationship was also significant among participants with non-DM, hypertension, and hyperlipidemia, as well as among non-smokers and alcohol consumers. Subgroup analyses did not detect any significant interactions between MPF intake and gallstones incidence among the analyzed subgroups (*p* for interaction > 0.05).

**TABLE 3:** Subgroup analyses corresponding to the MPF consumption and gallstones.

Variable	OR (95% CI)	*p*-Value	*p* for interaction
Age			0.08
<60	0.13 (0.04–0.41)	<0.001	
≥60	0.52 (0.20–1.33)	0.17	
Sex			0.38
Female	0.26 (0.12–0.55)	<0.001	
Male	0.57 (0.12–2.73)	0.47	
Race			0.84
Mexican American	0.24 (0.02–2.24)	0.20	
Non-Hispanic Black	0.25 (0.03–1.84)	0.17	
Non-Hispanic White	0.54 (0.18–1.56)	0.25	
Other Hispanic	0.22 (0.03–1.59)	0.13	
Other race	0.48 (0.11–2.03)	0.31	
Educational status			0.07
Less than high school	0.04 (0.00–0.51)	0.01	
High school	0.83 (0.32–2.11)	0.68	
More than high school	0.34 (0.13–0.87)	0.02	
BMI			0.99
<25	0.55 (0.06–4.89)	0.58	
25–30	0.51 (0.10–2.65)	0.41	
>30	0.59 (0.22–1.56)	0.28	
DM			0.38
No	0.37 (0.16–0.85)	0.02	
Yes	0.67 (0.23–1.99)	0.46	
Hypertension			0.87
No	0.45 (0.15–1.32)	0.14	
Yes	0.40 (0.18–0.90)	0.03	
Hyperlipidemia			0.11
No	0.81 (0.25–2.64)	0.72	
Yes	0.27 (0.13–0.57)	<0.001	
Smoke			0.14
No	0.31 (0.15–0.65)	0.003	
Yes	1.79 (0.22–14.59)	0.58	
Drinking			0.52
No	0.66 (0.07–6.09)	0.71	
Yes	0.30 (0.12–0.71)	0.01	

OR, odds ratio; CI, confidence interval; BMI, body mass index; DM, diabetes mellitus.

### 3.4 Mediation analyses

**FIGURE 3:**
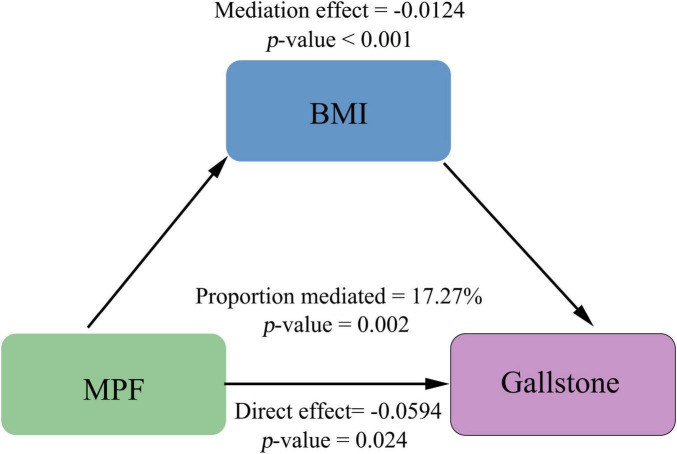
The mediating role of BMI in the association between MPF consumption and gallstones.

To further elucidate the mechanisms underlying the link between MPF intake and gallstones, we performed mediation analysis using non-parametric bootstrapping (1,000 simulations), with BMI as the hypothesized mediating variable. Our results demonstrate that BMI partially mediates the association between MPF intake and gallstones (Figure 3). The significant total negative association between higher MPF intake and gallstones (total effect = −0.0718, *p* = 0.002) comprised both a significant direct effect (average direct effect = −0.0594, *p* = 0.024) and a significant indirect effect mediated by BMI (average causal mediation effect = −0.0124, *p* < 0.001). The proportion of the total effect mediated by BMI was 17.27% (*p* = 0.002), indicating partial mediation.

## 4 Discussion

In this nationally representative cohort of the U.S. adult population, higher consumption of MPF was associated with reduced odds of gallstones. On the other hand, no statistically significant associations were observed between consumption of other NOVA food group, including UPF, and gallstones after controlling for potential confounding factors. Further analysis using RCS suggested a potential non-linear relationship between MPF consumption and gallstones. Additionally, mediation analysis revealed that BMI significantly mediated the association between MPF consumption and gallstones.

Minimally processed foods refer to natural foods that undergo limited processing without added ingredients. These foods may be subjected to basic techniques like grinding, heating (e.g., boiling or pasteurization), cooling (e.g., refrigeration or freezing), or non-alcoholic fermentation. Common examples include whole grains, legumes, fresh produce, animal products (meat, fish, eggs, and milk), and pure fruit juices, which serve as vital sources of endogenous antioxidant compounds (21, 22). Moreover, numerous processing techniques can lower the mean total antioxidant content for a food item. Processed fruits are associated with a lower antioxidant content (23). Moist-heat treatment (steaming) was found to markedly reduce the contents of lutein and β-carotene, well-characterized antioxidant micronutrients (24). Dry-heat application (roasting) resulted in significant decomposition of endogenous antioxidant compounds (25). Cooking, baking, and boiling vegetables decrease levels of vitamin C, phenolic compounds, and lycopene (26). While some cooking methods mentioned above are considered minimally processed, these findings also indicate that food processing may disrupt the intrinsic food matrix and diminish antioxidant levels. Emerging evidence indicated that total antioxidant content would be highest in MPF and lowest in UPF, according to the NOVA classification (21). Oxidative stress is firmly established as a regulator of gallstones development (12, 27, 28). The depletion of endogenous antioxidants through dietary processing methods may potentially exacerbate gallstones formation risk. Insufficient vitamin C intake may elevate gallstones risk by impairing free radical regulation, subsequently altering biliary protein-lipid composition and promoting stone formation (29–31). Evidence suggests that antioxidant-rich diets can help abrogate gallstones risk (32, 33). Therefore, the findings related to MPF consumption and gallstones may be partially explained by the high total antioxidant content of MPF.

Moreover, the observed inverse correlation between MPF consumption and gallstones formation may also be partially attributed to the anti-inflammatory properties of MPF. MPF serve as the foundation of several traditional healthy diets, such as the Mediterranean and Nordic diets, both of which are recognized for their anti-inflammatory properties (34, 35). Multiple meta-analyses evaluating dietary patterns on inflammatory indicated that higher adherence to the Mediterranean diet was correlated with reduced levels of inflammatory biomarkers, including C-reactive protein (CRP) and interleukin-6 (IL-6) (36). The Nordic diet has also demonstrated similar benefits, with evidence from intervention and observational studies highlighting its role in mitigating low-grade inflammation (37). The anti-inflammatory effects of these healthy diets may stem from their ability to enhance intestinal barrier integrity and modulate gut microbiota, thereby attenuating systemic inflammatory responses (38). These effects may be attributed to the high dietary fiber content in MPF, which promotes the production of short-chain fatty acids (SCFAs) (39). SCFAs are known to regulate neuroimmuno-endocrine functions and are associated with reduced levels of CRP and plasma lipopolysaccharide, a marker of intestinal permeability linked to low-grade inflammation (40, 41). In contrast to MPF, existing studies suggest that UPF, the counterpart in the NOVA classification, were often associated with elevated levels of systemic inflammation (42, 43). An analysis of three prospective cohort studies indicates that higher intake of UPF, particularly sugar-sweetened and artificially sweetened beverages, is linked to an increased risk of gallstones disease (18). However, no significant association between other NOVA food group consumption, including UPF and gallstones was found in this study. These contrasting findings highlight the complexity of the relationship between food processing and gallstones disease. Therefore, longitudinal studies with larger sample sizes and more detailed dietary assessments are warranted to better understand the interplay between food processing, inflammation, and gallstones disease. The mechanisms by which bioactive dietary constituents influence biliary metabolic processes through orchestrated modulation of bile acid synthesis, cholesterol solubilization dynamics, and enterohepatic signaling networks merit further exploration.

Given the role of obesity as a risk factor for gallstone, we further explored the mediating effect of BMI on the association between MPF intake and gallstones (1). A greater proportion of energy intake from MPF showed an inverse relationship with adiposity measures, including BMI, waist-to-height ratio (WHtR), and sagittal abdominal diameter-to-height ratio (SADHtR) (44). In contrast, epidemiological evidence indicated that higher consumption of UPF is associated with increased BMI in the population (45–47). The inverse relationship between MPF and obesity may be attributed to their high fiber and nutrient content, which enhances satiety and reduces total energy intake, thereby decreasing UPF consumption (44). In contrast, UPF are energy-dense, high in added sugars and fats, and low in essential nutrients, potentially promoting adiposity (48). Moreover, emerging evidence indicated that obesity is associated with changes in gastrointestinal hormones, which may contribute to gallstone development (49). Consequently, MPFs may counteract the adverse effects of UPFs on fat accumulation, contributing to a lower risk of obesity. The mediation analysis of our study confirmed BMI as a mediating variable in the relationship between MPF intake and gallstones. We observed a significant average causal mediation effect (*p* < 0.001), indicating that a portion of the protective effect of MPF intake against gallstones operates through reducing BMI. This mediated pathway accounted for 17.27% (*p* = 0.002) of the total effect. Importantly, a significant average direct effect (*p* = 0.024) was also observed, indicating that a substantial portion of the protective association operates through pathways independent of BMI.

The major strength of the present study lies in its use of a nationally representative, multiethnic cohort of U.S. adults, revealing a significant inverse association between MPF consumption and gallstones. These findings provide novel insights into the relationship between food processing and gallstones risk. To our knowledge, this is the first study to report an association between MPF consumption and gallstones in adults. Nonetheless, our study has several limitations. First, dietary intake was assessed using 24-h recalls, which are prone to recall bias and do not reflect long-term dietary patterns. Second, although food products were classified into the most likely NOVA group, individual-level misclassification cannot be entirely ruled out due to variations in processing methods across brands. Third, the cross-sectional design limits our ability to establish causal relationships. Fourth, the diagnosis of gallstones relied on participant self-reports, which may have led to recall bias and potential outcome misclassification. Fifth, while we adjusted for many potential confounders, residual confounding from factors such as hematological diseases, genetic predisposition to gallstone formation, prior bypass surgery, and other unmeasured variables not included in the NHANES database cannot be ruled out. Finally, as with any observational study, reverse causation remain potential concerns, as dietary changes following gallstones diagnosis may attenuate the observed associations.

## 5 Conclusion

In conclusion, these findings indicate that higher MPF intake is associated with a lower risk of gallstones disease, with BMI partially mediating this relationship (mediation proportion: 17.27%). However, no significant links were found between other NOVA food groups, including UPF, and gallstones. These results highlight the critical role of food processing in modulating gallstones formation, though further studies are warranted for confirmation. Our findings align with existing public health guidelines advocating for reduced UPF consumption and increased intake of MPF to promote overall health.

## Data Availability

Publicly available datasets were analyzed in this study. This data can be found here: https://www.cdc.gov/nchs/nhanes.
