# Supplementary material for: The mediating role of body mass index in the association between unprocessed or minimally processed foods and gallstones
Source: Front Nutr. 2025 Jun 23;12:1589805. doi: 10.3389/fnut.2025.1589805 (PMC12229848; doi:10.3389/fnut.2025.1589805)
Supplement: Supplementary file 1 [file Table_1.docx]

# Supplementary Table 1: Association of the PCI, PF, and UPF consumption and gallstone by logistic regression.

|  | **Model 1** | | **Model 2** | | **Model 3** | |
| --- | --- | --- | --- | --- | --- | --- |
|  | OR (95% CI) | *p*.value | OR (95% CI) | *p*.value | OR (95% CI) | *p*.value |
| PCI (% of total energy intake) | 0.17(0.02,1.63) | 0.12 | 0.11(0.01,1.75) | 0.11 | 0.04(0.00,2.79) | 0.13 |
| PF (% of total energy intake) | 1.76(1.18,2.64) | 0.01 | 1.54(0.98,2.43) | 0.06 | 1.71(0.96,3.07) | 0.07 |
| UPF (% of total energy intake) | 0.83(0.58,1.19) | 0.31 | 1.21(0.81,1.80) | 0.35 | 0.97(0.57,1.68) | 0.92 |

PCI, processed culinary ingredients; PF, processed foods; UPF, ultra-processed foods.
